# Prevalence of airway patency and air pocket in critically buried avalanche victims - a scoping review

**DOI:** 10.1186/s13049-024-01205-1

**Published:** 2024-04-23

**Authors:** Frederik Eisendle, Simon Rauch, Bernd Wallner, Hermann Brugger, Giacomo Strapazzon

**Affiliations:** 1 Institute of Mountain Emergency Medicine, Eurac Research, Via Ipazia 2, Bolzano, 39100 Italy; 2 Department of Anaesthesiology and Intensive Care Medicine, Medical University of Innsbruck, Innsbruck, Austria; 3 Department of Anaesthesia and Intensive Care Medicine, Hospital of Merano, Merano, Italy; 4 SIMeM Italian Society of Mountain Medicine, Padova, Italy

**Keywords:** Avalanche, Airway patency, Air pocket, Asphyxia, Outcome, Prevention

## Abstract

**Introduction:**

Survival of critically buried avalanche victims is directly dependent on the patency of the airway and the victims’ ability to breathe. While guidelines and avalanche research have consistently emphasized on the importance of airway patency, there is a notable lack of evidence regarding its prevalence.

**Objective:**

The aim of this review is to provide insight into the prevalence of airway patency and air pocket in critically buried avalanche victims.

**Methods:**

A scoping review was done in accordance with the Preferred Reporting Items for Systematic Reviews and Meta-Analyses (PRISMA) guideline extension for scoping reviews. MEDLINE and Cochrane databases, as well as additional manual searching was performed to identify literature reporting data on airway patency and the presence of an air pocket in critically buried avalanche victims. After eliminating duplicates, we screened abstracts and main texts to identify eligible studies.

**Results:**

Of 4,109 studies identified 154 were eligible for further screening. Twenty-four publications and three additional data sources with a total number of 566 cases were included in this review. The proportion of short-term (< 35 min) to long-term burial (≥ 35 min) in the analysed studies was 19% and 66%, respectively. The burial duration remained unknown in 12% of cases. The prevalence of airway patency in critically buried avalanche victims was 41% while that of airway obstruction was 12%, with an overall rate of reporting as low as 50%. An air pocket was present in 19% of cases, absent in 46% and unknown in 35% of the cases.

**Conclusion:**

The present study found that in critically buried avalanche victims patent airways were more than three times more prevalent than obstructed, with the airway status reported only in half of the cases. This high rate of airway patency supports the ongoing development and the effectiveness of avalanche rescue systems which oppose asphyxiation in critically buried avalanche victims. Further effort should be done to improve the documentation of airway patency and the presence of an air pocket in avalanche victims and to identify factors affecting the rate of airway obstruction.

**Supplementary Information:**

The online version contains supplementary material available at 10.1186/s13049-024-01205-1.

## Introduction

Survival of critically buried avalanche victims (defined as head and chest completely covered by snow) is directly dependent on the patency of the patients’ airway and their ability to breathe [1–3]. Airway patency is defined as mouth and nose free from snow or other obstructions of the respiratory tract in critically buried avalanche victims [3–5]. The ability to breathe depends on the access to fresh air through the presence of an air pocket or low snow density [6], and the possibility of chest expansion.

No cases of survival with obstructed airway after burial ≥ 35 min are reported in literature and the presence of an air pocket is associated with increased survival [7]. Even though established guidelines and existing literature in avalanche research highlight the importance and necessity for assessing airway patency in avalanche victims [3, 8], there is still a substantial lack of evidence on the prevalence of obstructed airways and presence of an air pocket, especially for short burial times (< 35 min). This scarcity can be due to the difficulties in the assessment during extrication, the lack of on-scene health care providers at the moment of extrication [9], as well as the current recommendation to assess it only for long burial times [3–5]. This contributes to a documentation and reporting bias in particular for short-term burials.

Personal preventive and protective avalanche equipment aims to reduce mortality among critically buried avalanche victims, avoiding asphyxia by channelling exhaled carbon dioxide away from the airway and providing fresh air from the surrounding snow to the critically buried victim [5, 10–12]. Hence, the knowledge of prevalence of airway patency and the presence of an air pocket is of major interest for the triage and treatment of critically buried avalanche victims and gains paramount importance when assessing and comparing the overall effectiveness of these devices in its purpose of increasing survival.

This scoping review aimed to give an overview on the prevalence of airway patency and the presence of an air pocket in critically buried avalanche victims and their impact on survival.

## Methods

This study adhered to the Preferred Reporting Items for Systematic Reviews and Meta-Analysis (PRISMA– www.prisma-statement.org) guideline extension for scoping reviews [13].

### Eligibility criteria

Studies in either English, German or Italian language, published from 1985 to 2023 were included. All studies related to avalanches involving victims were considered eligible for further review. Studies were excluded if they did not address snow avalanches or if they did not report information on airway patency. Only cases of critical burial were considered for final analysis. Studies regarding snow immersion were excluded.

### Search strategy

MEDLINE database was searched via PubMed using the search terms “avalanche” and “snow AND airway”. The Cochrane Database for Systematic Reviews was searched for the term “avalanche” in Title, Abstract or Keywords. Additional manual searching of reviewed articles, reference texts and reference lists for relevant studies was performed. Search was conducted in July 2023.

### Outcomes, data extraction and analysis

Primary outcome measure was prevalence of patent versus obstructed airway for short and long burials reported in percentage of cases. Burial duration was defined as short if < 35 min and long if ≥ 35 min, as no victims were reported to have survived burials longer than 35 min with an obstructed airway [7, 14]. Secondary outcome measures were the rates of a present air pocket and survival in percentage of cases, both in relation to burial time and airway status.

One reviewer (FE) extracted the data from the selected studies. Identified literature was sought for data reporting patients’ airway status (defined as patent, obstructed or unknown) after critical burial. Additionally, studies were screened for burial duration, mortality and information on the presence of an air pocket which was included in the final analysis of secondary outcomes. Some studies further categorised data on airway status as “not documented, but intubated or ventilated” or “not documented and not intubated/ventilated”, these cases were added to the unknown airway category.

Raw data was used for data extraction where available. Data was charted in tables for every category and publication (separated for retrospective studies, case reports, and additional databases) in Microsoft Excel (Microsoft Corporation, Redmond, USA) and analysed with SPSS statistics software version 28.0. (IBM Corp., Armonk, USA). Odds ratio (OR) was calculated to assess the correlation between airway patency, the presence of an air pocket and survival.

## Results

**Fig. 1:**
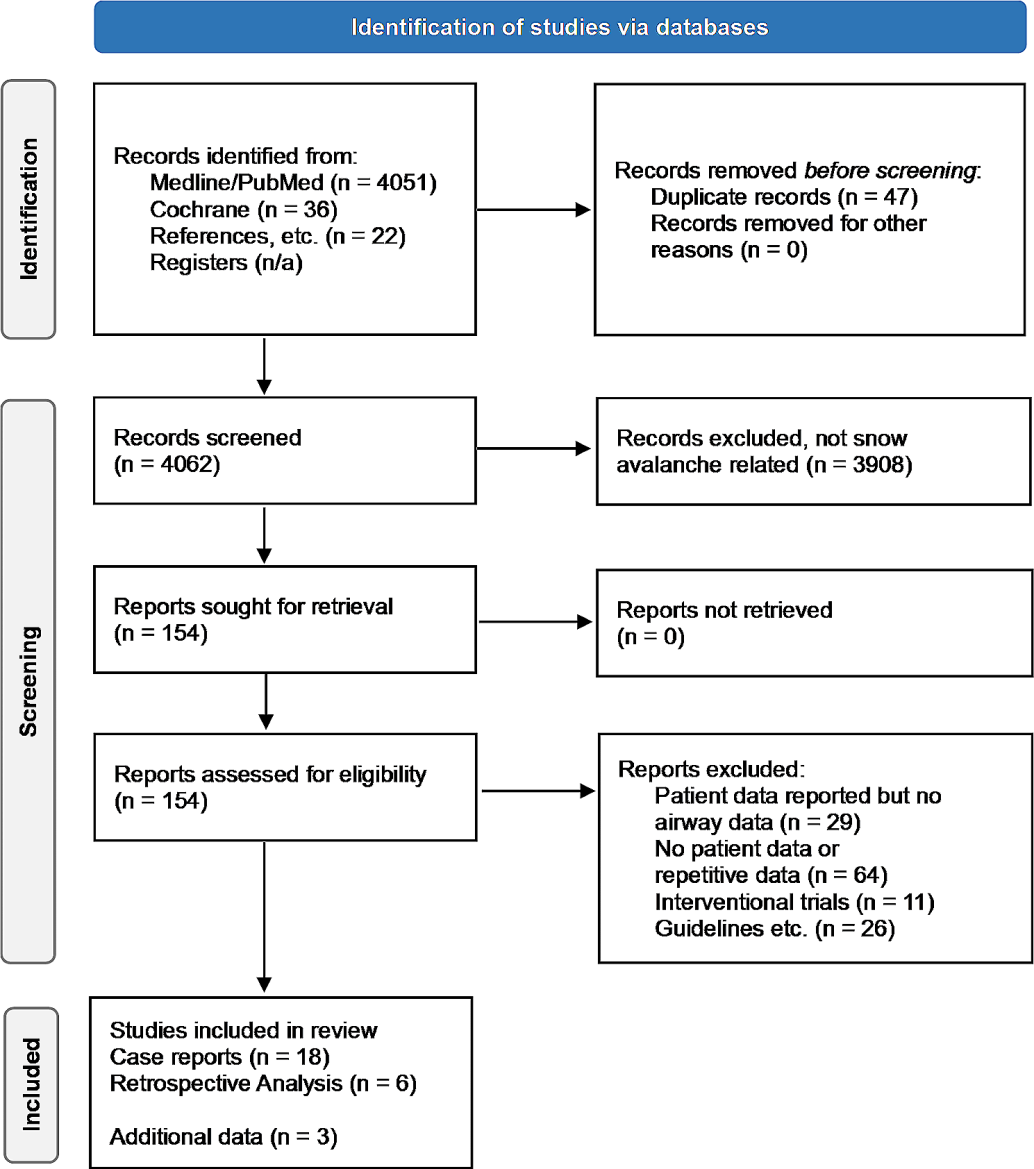
PRISMA flow diagram of conducted search

A total of 4,109 studies were identified from databases, out of these 47 duplicates which were excluded. Further 3,908 studies were excluded because they were not primarily related to snow avalanches. The remaining 154 studies were further evaluated for eligibility.We rejected 130 studies which failed to meet inclusion criteria. Our search finally identified 24 studies (Fig. 1), of which 6 had a retrospective data analysis design (Supplemental Table 1) [15–20] and 18 case reports (Supplemental Table 2) [21–38]. Additional data on airway patency was available from internal data repository for two of the previous retrospective studies [16, 18] and one master thesis (Supplemental Table 3) [39].

A total of 566 cases was included in the primary analysis. The variable “air pocket” could not be matched with “burial times” in two studies including 158 cases (Blancher et al. [17] and Eidenbenz et al. [20]). “Survival” variable could not be matched with “burial times” in one study including 18 cases (Blancher et al. [17]). Cases of Métrailler-Mermoud et al. [19] were not used for the survival analysis for possible selection bias.

All retrospective studies report data on airway patency (Table 1). Boué et al. [15] reported 48 cases, two (4%) cases with obstructed airway. Hence, all of remaining 46 (96%) cases were counted as patent airways. Strapazzon et al. [16] reported data on airway patency for long term burials. Of the 108 reported cases, 59 (55%) cases showed airway obstruction, while 23 (21%) of cases had a patent airway. Airway status of the remaining cases (26 patients, 24%) was unknown. Additional data on airway patency for 34 short term burial cases were available from the data repository of this study (Table 2). Blancher et al. [17] reported an interesting multicausality incident with 18 critically buried subjects with 33% of obstructed airways and 66% with patent airways. Brugger et al. [18] investigated avalanche victims (*n* = 61) treated with extracorporeal rewarming in multicentre data study from 1995 to 2016. This represents data from a highly selected subpopulation but additional data on airway patency was available from data repository by Brugger et al., reporting additional 42 cases which were not rewarmed by active invasive measures (Table 2). To minimize the risk of potential selection bias for this study, we used the complete dataset comprising all available cases (*n* = 103) for our analysis. Métrailler-Mermoud et al. [19] reported 59 cases of non-survivors after critical burial, of which 2 (3%) with obstructed and 11 patients (19%) with patent airways. Airway status for 78% of cases was unknown. Eidenbenz et al. [20] reported airway patency for long-term burials over 60 min for 140 patients. 20 victims (14%) had patent airways, 16 (12%) were obstructed. The number of unknown airway status was 104 (74%). Internal data repository reported data for 35 cases from a master thesis [39] (Table 2). Eighteen case reports reported data for 11 survivors and ten deceased. The overall prevalence of obstructed airway was 19%, while the one of patent airway was 81% (Table 3).

**Table 1:** Analysis of retrospective data for airway patency (not assigned: reported, but not in relation to burial time)

		Obstructed	Patent	Unknown
Burial time	Short < 35 min (*n* = 23, 5%)	0	5 (22%)	18 (78%)
	Long ≥ 35 min (*n* = 271, 63%)	37 (14%)	40 (15%)	194 (71%)
	Unknown (*n* = 61, 14%)	2 (3%)	48 (79%)	11 (18%)
	Not assigned (*n* = 79, 18%)	8 (10%)	58 (73%)	13 (17%)
Total (*n* = 434)		47 (11%)	151 (35%)	236 (54%)

**Table 2:** Analysis of available additional raw data for airway patency

		Airway		
		Obstructed	Patent	Unknown
Burial time	Short < 35 min (*n* = 63, 57%)	12 (19%)	26 (41%)	25 (40%)
	Long ≥ 35 min (*n*= 41, 37%)	6 (15%)	15 (37%)	20 (48%)
	Unknown (*n* = 7, 6%)	0 (3%)	4 (57%)	3 (43%)
Total (*n* = 111)		18 (16%)	45 (41%)	48 (43%)

**Table 3:** Analysis of case reports for airway patency

		Obstructed	Patent	Unknown
Burial time	Short < 35 min (*n* = 8, 38%)	2 (25%)	6 (75%)	0
	Long ≥ 35 min (*n* = 13, 62%)	2 (15%)	11 (85%)	0
Total (*n* = 21)		4 (19%)	17 (81%)	0

### Overall analysis

The proportion of short-term to long-term burials in the analysed studies was 19% and 66%, respectively.

The duration of burial remained unknown in 15% of the cases. The overall rate of reporting airway status was 50% (ranging from 0 to 78%). The reporting rate of airway patency was lower compared the presence of an air pocket (65% of cases) and the survival status (78% of cases).

#### Airway patency

The mean rate of patent airways was 38% (ranging from 14 to 96%) in this sample of 566 cases. The prevalence of obstructed airway was 12% (ranging from 4 to 55%) (Table 4). The 19 (3%) cases who survived long burial were additionally attributed to the patent airway category, since survival in long term burial is only possible with a patent airway, resulting in a final number of an expected patent airway of 232 (41%) (Table 5).

**Table 4:** Overall analysis for airway patency in 566 cases (not assigned: reported, but not in relation to burial time)

		Obstructed	Patent	Unknown
Burial time	Short < 35 min (*n* = 106, 19%)	15 (14%)	43 (41%)	48 (45%)
	Long ≥ 35 min (*n* = 374, 66%)	46 (12%)	106 (29%)	222 (59%)
	Unknown (*n* = 68, 12%)	2 (3%)	52 (77%)	14 (20%)
	Not assigned (*n* = 18, 3%)	6 (33%)	12 (67%)	0
Total (*n* = 566)		69 (12%)	213 (38%)	284 (50%)

**Table 5:** Overall analysis for the presence of an air pocket in comparison to burial time and airway status (selected retrospective studies, case reports and additional data, 158 non-matchable cases excluded)

			Air pocket				
Burial time			Absent	Present	Unknown	Total	
Short (< 35 min)	Airway	Obstructed	14 (93%)	0	1 (7%)	15	
		Patent	12 (28%)	15 35%)	16 (37%)	43	
		Unknown	11 (23%)	1 (2%)	36 (75%)	48	
	Total		37 (35%)	16 (15%)	53 (50%)	106 (26%)	
Long (≥ 35 min)	Airway	Obstructed	28 (93%)	0	2 (7%)	30	
		Patent	33 (38%)	42 (49%)	11 (13%)	86	
		Unknown	43 (36%)	12 (10%)	64 (54%)	118	
	Total		104 (44%)	54 (23%)	76 (33%)	234 (57%)	
Unknown	Airway	Obstructed	2 (100%)	0	0	2	
		Patent	42 (81%)	7 (13%)	3 (6%)	52	
		Unknown	1 (7%)	0	13 (93%)	14	
	Total		45 (66%)	7 (10%)	16 (24%)	68 (17%)	
Total	Airway	Obstructed	44 (94%)	0	3 (6%)	47	
		Patent	87 (48%)	64 (35%)	30 (17%)	181	
		Unknown	55 (31%)	13 (7%)	112 (62%)	180	
	Total		186 (46%)	77 (19%)	145 (35%)	408	

#### Air pocket

An air pocket was reported in 77 (19%) of the 408 cases, while it was absent in 186 (46%) of the cases; it was not reported in 145 (35%) cases (Table 6). All cases with an obstructed airway were associated with the absence of an air pocket. Patent airways without additional air pocket were reported in 87 (48%) cases.

**Table 6:** Overall analysis for survival in comparison to burial time and airway status (selected retrospective studies, case reports and additional data, 18 non-matchable cases and cases of Métrailler-Mermoud et al. [19] excluded)

			Survival				
Burial time			Died	Survived	Unknown	Total	
Short (< 35 min)	Airway	Obstructed	6 (40%)	1 (7%)	8 (53%)	15	
		Patent	17 (45%)	12 (31%)	9 (24%)	38	
		Unknown	15 (50%)	3 (10%)	12 (40%)	30	
	Total		38 (46%)	16 (19%)	29 (35%)	83 (17%)	
Long (≥ 35 min)	Airway	Obstructed	27 (61%)	1 (2%) *	16 (37%)	44	
		Patent	65 (64%)	25 (25%)	12 (11%)	102	
		Unknown	123 (60%)	19 (9%)	63 (31%)	205	
	Total		215 (61%)	45 (13%)	91 (26%)	351 (72%)	
Unknown	Airway	Obstructed	2 (100%)	0	0	2	
		Patent	38 (76%)	12 (24%)	0	50	
		Unknown	2 (67%)	1 (33%)	0	3	
	Total		42 (76%)	13 (24%)	0	55 (11%)	
Total	Airway	Obstructed	35 (57%)	2 (3%)	24 (40%)	61	
		Patent	120 (63%)	49 (26%)	21 (11%)	190	
		Unknown	140 (59%)	23 (10%)	75 (31%)	238	
	Total		295 (63%)	74 (14%)	120 (23%)	489	

* = An error in understanding of the term “patent airway” by the emergency physician is suspected in this case, in which nasal oxygen therapy was administered during transport [19]

#### Survival

Overall mortality was 63% for the 489 documented cases and 74 victims (14%) survived; outcome data was missing in 120 cases (23%) (Table 5). Cases of Métrailler-Mermoud et al. [19] were not included in survival analysis to avoid potential selection bias. Victims with obstructed airway were more likely to die (OR = 7.1, 95% CI [1.65, 30.87], *p* = 0.008) compared to those presenting with a patent airway. Victims with a patent airway without air pocket were also more likely to die than those with patent airway and a present air pocket (OR = 4.7, 95% CI [1.43, 15.19], *p* < 0.01) (Supplemental Table 4).

## Discussion

This review investigated the overall prevalence of airway patency and presence of an air pocket in critically buried avalanche victims in the literature. An overall rate of 41% was found for a patent airway in critically buried avalanche victims, while 12% had an obstructed airway. The overall reporting rate for airway status was low with 50% of cases where this information was not reported. An air pocket was present in 19% of cases, while it was absent in 46% of the cases. The presence of an air pocket was not reported in 35% of the cases.

About 75% of critically buried victims die of asphyxia due to an obstructed airway or an excess of carbon dioxide in the inhaled air [3, 7, 40]. The terms obstructed or blocked airway require that both, mouth and nose are completely obstructed with compacted snow debris and the obstruction can be caused by snow or vomitus. Different factors affect the rate of obstruction, such as different avalanche dimensions, avalanche debris characteristics, phase of ascent or descent. To our knowledge, those factors have not been analysed yet. No reported avalanche victim with an obstructed airway has survived a long-term burial exceeding 35 min [3, 14, 41]. This led to the causal conclusion that avalanche victims who survived a critical burial of ≥ 35 min must have had patent airways. Conversely, some short-term burials even with obstructed airway may have survived with little or no serious consequences on their health, if the victim was rescued in less than 15–18 min [2, 7]. However, this is not necessarily the case for all short-term burials as traumatic injuries or respiratory failure may occur after a few minutes of critical burial.

Airway patency is a cornerstone in the triage and management of critically buried avalanche victims in the guidelines of International Liaison Committee on Resuscitation (ILCOR), of the International Commission for Alpine Rescue (ICAR), and of the Wilderness Medical Society (WMS) [3, 5, 42]. Despite the clear emphasis on the importance of evaluation of airway patency and the presence of airway pocket, this information is only reported seldomly and missing in half of all cases documented in the literature.

Recommendations for on-site treatment of critically buried avalanche victims advise to always presume asphyxia as the primary cause for non-traumatic cardiac arrest in victims with a burial time < 60 min and provide rescue breaths as soon as possible, regardless of airway patency. They further suggest determining the airway status when the face is exposed only if the burial time is > 60 min [3]. This may contribute to a documentation and reporting bias of airway status in short term burials.

Our current findings are particularly relevant for preventive and management strategies in increasing survival. Though avalanche airbags and other preventive strategies have shown to be efficient in increasing survival probability in avalanche victims [5, 43, 44], the risk of critical burial persists [45, 46]. Artificial air-pocket devices (AAPD) are intended to enable critically buried victims to delay asphyxia by channelling exhaled carbon dioxide away from the airways and may also offer airway protection by using a mouthpiece [10, 11]. The usage and overall effectiveness of artificial air-pocket devices is still in question [46, 47], mostly due to the concern on the capability to insert the mouthpiece during the cause of avalanche burial. The efficacy of a new system which also aims to reduce the re-breathing of carbon-dioxide-rich exhaled air by providing fresh air without the need of a mouthpiece, is currently under investigation in a randomised clinical trial [48]. 

The presence of an air pocket is associated with increased chances for survival and a better neurological outcome [7]. Up until 2021, the assessment of the air pocket was not included in the algorithm for triage and management of avalanche victims [41, 49, 50], our data show that it was nonetheless reported with a higher frequency than the airway status (65% vs. 50% of the cases).

The present study supports the existing understanding that survival in critically buried victims is relatively low and that maintaining airway patency is crucial for survival. Survival of long-term burials is not possible without a patent airway [7]. 

### Limitations

Studies show a high percentage of missing data and the risk of relative bias, which may be the biggest limitations of this study. Specifically, a substantial portion of data from the included studies carry a notable risk of reporting bias, primarily due to its tendency of underreporting short-term burials with positive outcome. This might explain our study’s 63% overall mortality rate that is higher compared to previous findings [3, 40, 41]. 

Additionally, this high mortality could be attributed to the study’s focus on including only studies with documented data on airway status, predominantly from hospitalised patients. Consequently, this study may overlook a significant number of especially short-term critically buried avalanche victims without the need of medical treatment, leading to unrecorded cases with generally good survival chances. As some studies refer only to patients transported to the hospital, there is also a risk of underreporting obstructed airways for patients considered dead on site.

Finally, this scoping review was limited to articles in English, German or Italian language. However, search identified no articles in other languages. Raw data was not available for all identified studies.

## Conclusions

The prevalence of airway patency in critically buried avalanche victims was 41% while that of airway obstruction was 12%, with an overall rate of reporting as low as 50%. The reporting rate of airway patency is as low as 50%. The high rate of airway patency supports the ongoing development and the effectiveness of avalanche rescue systems strategies which oppose asphyxiation in critically buried avalanche victims. Further effort should be undertaken to improve the documentation of airway patency and the presence of an air pocket in avalanche victims, and to identify factors affecting the rate of airway obstruction.

### Electronic Supplementary Material

Below is the link to the electronic supplementary material.

Supplemental tables

## Data Availability

The datasets used and/or analysed during the current study available from the corresponding author on reasonable request.
