# Supplementary material for: Prevalence of airway patency and air pocket in critically buried avalanche victims - a scoping review
Source: Scand J Trauma Resusc Emerg Med. 2024 Apr 23;32:34. doi: 10.1186/s13049-024-01205-1 (PMC11040957; doi:10.1186/s13049-024-01205-1)
Supplement: Supplementary file 1 — Supplemental tables [file 13049_2024_1205_MOESM1_ESM.docx]

Supplemental tables

| **Title** | **Author (Ref.)** | **Year** | **Cases** | **Raw data available** |
| --- | --- | --- | --- | --- |
| Survival after avalanche-induced cardiac arrest | Boué et al. (21) | 2014 | 48 | yes |
| Prehospital management and outcome of avalanche patients with out-of-hospital cardiac arrest: a retrospective study in Tyrol, Austria | Strapazzon et al. (15) | 2017 | 108 | yes* |
| Multiple casualty incident in the mountain: Experience from the Valfrejus avalanche | Blancher et al. (18) | 2017 | 18 | no |
| Cut-off values of serum potassium and core temperature at hospital admission for extracorporeal rewarming of avalanche victims in cardiac arrest: A retrospective multi-centre study | Brugger et al. (16) | 2019 | 61 | yes* |
| Avalanche victims in cardiac arrest are unlikely to survive despite adherence to medical guidelines | Métrailler-Mermoud et al. (22) | 2019 | 59 | yes |
| Survival probability in avalanche victims with long burial (>60 min): A retrospective study | Eidenbenz et al. (19) | 2021 | 140 | no |
| Total number of cases | | | 434 |  |

Supplemental table 1: Identified studies with retrospective design (* = additional data available, see Supplemental table 3)

| **Title** | **Author (Ref.)** | **Year** | **Cases** |
| --- | --- | --- | --- |
| Survival after burial in an avalanche | Gray et al. (36) | 1987 | 1 |
| Full recovery of an avalanche victim with profound hypothermia and prolonged cardiac arrest treated by extracorporeal re-warming | Oberhammer et al. (37) | 2008 | 2 |
| Cooling of six centigrades in an hour during avalanche burial | Putzer et al. (38) | 2010 | 1 |
| Respiratory Failure and Spontaneous Hypoglycemia During Noninvasive Rewarming From 24.7°C (76.5°F) Core Body Temperature After Prolonged Avalanche Burial | Strapazzon et al. (39) | 2012 | 1 |
| Lebensrettender luftgestützter Lawineneinsatz bei Nacht im hochalpinen Gelände (Life-saving air supported avalanche mission at night in high alpine terrain) | Koppenberg et al. (40) | 2012 | 2 |
| Electrical Heart Activity Recorded During Prolonged Avalanche Burial | Strapazzon et al. (41) | 2012 | 1 |
| Electrical cardiac activity in an avalanche victim dying of asphyxia | Heschl et al. (42) | 2013 | 1 |
| Full Neurologic Recovery after Prolonged  Avalanche Burial and Cardiac Arrest | Boué et al. (43) | 2014 | 2 |
| Mechanical Chest Compressions in an Avalanche Victim With Cardiac Arrest: An Option for Extreme Mountain Rescue Operations | Pietsch et al. (44) | 2014 | 1 |
| Cooling rate of 9.4°C in an hour in an avalanche victim | Pasquier et al. (45) | 2015 | 1 |
| Apparent Cooling Rate of 7°C per Hour in an Avalanche Victim | Ströhle et al. (46) | 2015 | 1 |
| Extracorporeal membrane oxygenation in avalanche victim with deep hypothermia and circulatory arrest | Facchetti et al. (47) | 2016 | 1 |
| Negative pressure pulmonary oedema with haemorrhage after 5-minute avalanche burial | Glisenti et al. (48) | 2016 | 1 |
| The longest persisting ventricular fibrillation with an excellent outcome – 6 h 45 min cardiac arrest | Kosinski et al. (49) | 2016 | 1 |
| A case of successful organ donation after extremely prolonged manual cardiopulmonary resuscitation in an avalanche victim | Varutti et al. (50) | 2019 | 1 |
| Extreme Cooling Rates in Avalanche Victims: Case Report and Narrative Review | Mittermair et al. (51) | 2021 | 1 |
| Witnessed Cardiac Arrest in a Hypothermic Avalanche Victim Completely Buried for 2 Hours | Daniel et al. (52) | 2021 | 1 |
| Critically buried avalanche victims can develop severe hypothermia in less than 60 min | Rauch et al. (53) | 2023 | 1 |
| Total number of cases | | | 21 |

Supplemental table 2: Identified case reports

| **Title** | **Author** | **Year** | **Cases** |
| --- | --- | --- | --- |
| Prehospital management and outcome of avalanche patients with out-of-hospital cardiac arrest: a retrospective study in Tyrol, Austria | Strapazzon et al. (15) | 2017 | 34 |
| Cut-off values of serum potassium and core temperature at hospital admission for extracorporeal rewarming of avalanche victims in cardiac arrest:  A retrospective multi-centre study | Brugger et al. (16) | 2019 | 42 |
| Gestione dei pazienti travolti da valanga (Valle d’Aosta and Piemonte database) | Berteletti et al. (17) | 2016 | 35 |
| Total number of cases | | | 111 |

Supplemental table 3: Identified additional data from retrospective studies

| Airway status | | | **Survival** | | | |  |
| --- | --- | --- | --- | --- | --- | --- | --- |
|  |  |  | Deceased | Survived | Unknown | Total |  |
| Obstructed | Air pocket | Absent | 19 | 1 | 24 | 44 |  |
|  |  | Present | 0 | 0 | 0 | 0 |  |
|  |  | Unknown | 3 | 0 | 0 | 3 |  |
|  | Total | | 22 | 1 | 24 | 47 |  |
| Patent | Air pocket | Absent | 73 | 5 | 9 | 87 |  |
|  |  | Present | 29 | 23 | 12 | 64 |  |
|  |  | Unknown | 21 | 9 | 0 | 30 |  |
|  | Total | | 123 | 37 | 21 | 181 |  |
| Unknown | Air pocket | Absent | 14 | 1 | 40 | 55 |  |
|  |  | Present | 7 | 0 | 6 | 13 |  |
|  |  | Unknown | 79 | 4 | 29 | 112 |  |
|  | Total | | 100 | 5 | 75 | 180 |  |
| Total | Air pocket | Absent | 106 | 7 | 73 | 186 |  |
|  |  | Present | 36 | 23 | 18 | 77 |  |
|  |  | Unknown | 103 | 13 | 29 | 145 |  |
|  | Total | | 245 | 43 | 120 | 408 |  |

Supplemental table 4: Comparison of survival compared to airway status and presence of an air pocket. Cases of Métrailler-Mermoud et al. (22) excluded.
